# Managing the medical resources of a national insurance program: lessons based on China’s NCMS

**DOI:** 10.1186/s12939-022-01694-5

**Published:** 2022-07-11

**Authors:** Wenqiang Qian, Xiangyu Cheng

**Affiliations:** 1 School of Public Administration, China University of Geosciences, Wuhan, 430074 China; 2 The Co-Innovation Center for Social Governance of Urban and Rural Communities in Hubei Province, Zhongnan University of Economics and Law, Wuhan, 430073 China

**Keywords:** Intergovernmental transfer, Medical insurance fund balance, Strategic behavior

## Abstract

**Background:**

The security of medical insurance fund is very important to health equity. In China, the expenditure of medical insurance fund has increased sharply year after year, and the balance of local medical insurance fund is difficult to sustain. To realize the equitable distribution of the medical insurance burden, the central government has to continuously increase transfer payments, which causes regional unfairness in the distribution of central financial resources. This paper explores the influence of central transfer payments on the balance of medical insurance fund, influential mechanisms, and the strategic behavior of local governments.

**Methods:**

First, we constructed a dynamic game model between central government and local governments and analyzed the mechanism of central transfer payments affecting the balance of local medical insurance fund. Then, based on the provincial panel data of 28 provincial administrative regions in China from 2004 to 2014, an empirical test was made. The spatial regression model was constructed, and the transfer payments obtained by neighboring provinces in the previous year were taken as instrumental variables.

**Results:**

Central transfer payments led to strategic behaviors by local governments that resulted in increased local health insurance fund expenditures and lower balance rates. Moreover, the central transfer payments demonstrated “path dependence”. Central transfer payments had a significant negative influence on the local NCMS fund balance rate. The local government subsidy and per capita GDP had a significant positive impact on the local NCMS fund balance rate. The obtained transfer payments of local governments had a significant space correlation. This study based on NCMS data remains valid.

**Conclusions:**

Central transfer payments induced the strategic behavior of local governments, which neglected to supervise the expenditure of medical insurance fund, reducing the efficiency of medical insurance fund management and use. The financial resources of medical insurance fund are unevenly distributed among provinces. Measures such as strengthening the supervision ability and initiatives of local governments, refining the central transfer payment mechanism, promoting the economic growth of western regions, and increasing rates for individual contributions appropriately can ensure that the medical insurance fund are used well and distributed equitably.

## Background

In recent years, China’s medical insurance fund has grown rapidly. For instance, the expenditures of NCMS fund increased from RMB2.64 billion ($0.32 billion) in 2004 to RMB293.34 billion ($47.09 billion) in 2015. To realize the equality of health rights among different provinces, the central government provides a lot of transfer payments to local governments, especially the developing provinces. From 2004 to 2015, central transfer payments increased from RMB0.68 billion ($82 million) to RMB150.14 billion ($24.10 billion). The rapid growth of expenditure seriously threatens the sustainability of medical insurance fund. Therefore, it is necessary to explore the influence and possible mechanism of central transfer payments on the expenditures of local medical insurance fund.

Previous studies have focused on the impact of transfer payments on local government revenues and expenditures. They found that the central transfer payment will cause strategic responses of the local government, such as less effort on tax collection, and more local government expenditures [1–4]. On average, the expenditures or debts of local governments would increase by 20% as a result of central transfer payments [5]. And the more debts of the local governments, the more transfer payments they will get from the central government [6, 7]. In addition, transfer payments may lead to an “inefficient lock-in” of vertical fiscal imbalance [8].

However, few studies focus on the impact of transfer payments on the expenditures of medical insurance fund. The strategic behavior of the local government in medical insurance fund supervision almost has not been captured. The subsidies from the central government to local governments, like a safety net for local governments, make it possible to enable them to relax supervision, and increase medical insurance fund expenditures when they expect to obtain transfer payments from the central government [9–11]. As a result, the local government’s shortfall grows even larger. Thus, the central government has to increase transfer payments, especially to the provinces with slack supervision. Inequities in health care and resource distribution among different provinces have been aggravated [12, 13]. However, there is little evidence from China, although China has the world’s largest health care system.

In this study, we construct a dynamic central-local game model, that allows for the strategic behavior of local government, and theoretically analyze how central transfer payments affect the behaviors of local governments. Based on provincial-level NCMS^1^ data, we verify and demonstrate the theoretical analysis results. The primary theoretical contribution of this paper is to construct the response function of the local government's regulatory behavior to the central government's transfer payments and make an empirical test, clarifying the relationship mechanism between them. In addition, the research results will provide decision-making evidence for medical insurance fund transfer and the promotion of health equity.

The remainder of this paper is organized as follows. The authority and responsibility of different levels of government regarding the NCMS are introduced first. The “Methods” section constructs a dynamic central-local game model, analyzes how central transfer payments affect the behaviors of local governments theoretically, and describes the models and data origin. The “Results” section shows the empirical results. The “Discussion” section summarizes the findings and their implications. The last section presents the conclusions.

### Intergovernmental relationships in the NCMS

As the world's largest medical insurance system ever, NCMS was established in 2003. To guarantee that farmers’ basic medical needs are satisfied, alleviate their medical burdens, and address the problem of poverty caused by illness or prevent them from getting poor again because of illness, in 2002 the Chinese government began to set up a new rural cooperative medical service system^2^ based mainly on a financial-pool-against-serious-disease scheme. And then, in 2003, the General Office of the State Council forwarded the Opinions on Building of a New Type of Rural Cooperative System, which means that the NCMS was formally established. In 2016^3^ China officially integrated basic medical insurance for non-working urban residents and the new type of rural cooperative medical care, to unify insurance coverage, funding policies, insured treatment, reimbursement catalogs, management of contracted medical institutions, and fund management. Eventually, the NCMS was integrated into a unified basic medical insurance system for rural and non-working urban residents.

The NCMS is organized, led, and supported by the government. First, the state has issued several regulatory documents to guide the establishment of NCMS. Second, the funds needed are pooled from individual payments, collective support, and government subsidies, and more than half of the funds are government subsidies. In the beginning, the individual contribution standard should be no less than RMB10 per person per year, and the local government subsidies for farmer participants should be higher than RMB10 per capita. Since 2003, the central government has also provided subsidies of RMB10 per capita to farmer participants in the central and western regions (in addition to the downtown areas). Since 2013, when the central government proposed to “reasonably divide financing responsibilities of the government and individuals and make an appropriate increase of individual contributions while improving the subsidy standards of the government”, the proportion of government subsidies in the total funding has decreased to some extent but still amounts to more than 50%.

The subsidy from the central government varies in different regions. Upon the establishment of NCMS, the central government and the local government equally shared the responsibility of providing subsidies in the central and western regions, except in several provinces, such as Hebei and Hainan, but the central region provided no subsidy for most eastern regions. To expand NCMS’s coverage,^4^ the central government has also provided subsidies to participants of farmers in eastern China since 2006.

Generally, the central government only provided a small number of subsidies to the eastern region, and the local governments in the eastern region still assumed the primary financing responsibility. In 2018, the General Office of the State Council issued the Notice of Reforming Plan for Defining the Respective Fiscal Powers and Expenditure Responsibilities of Central and Local Governments in Health Care, which realigned financial subsidy responsibilities among the central and local governments for basic medical insurance for rural and non-working urban residents. The central government provides 80% of financial assistance for first-class regions, 60% for second-class regions, 50% for third-class regions, 30% for fourth-class regions, and 10% for Beijing and Shanghai.^5^ Then, the differences among regions in receiving subsidies from the central government become more obvious.

The government is comprehensively improving its financial subsidy based on its economic and social development. In 2009, the Health Ministry issued Opinions on Consolidating and Developing the New Type Rural Cooperative Medical Care System, which clearly states that the financial subsidy standard and farmers’ contribution shall be gradually increased according to the financial situation of governments at all levels and the income increase of farmers, respectively. Government subsidies for NCMS and per capita funding have grown year by year. The government subsidy standards for NCMS increased from RMB20 at the beginning to RMB580 per person per year in 2021, with the individual contribution rising to RMB320 per person per year. Different regions and provinces have different levels of economic development, and there is a significant difference in financial strength among provinces. To balance the financial burden of regions with different financial strengths, the central government has increased its subsidy to regions with lower levels of economic development and worse financial strength. For example, the central government provides 80% of the subsidy for Nei Mongol, an area of relatively weak financial strength, but only 10% for the major cities of Beijing and Shanghai.

Furthermore, the revenue and expenditures of the NCMS fund in the previous year also affect the government’s subsidy. In 2010 the Seventeenth Session of the Standing Committee of the Eleventh National People’s Congress promulgated the Social Insurance Law of the People’s Republic of China, which clearly states that the balanced budget is an intrinsic requirement of the social insurance fund. However, the balance of fund income and expenditure does not mean fund revenue is equal to fund expenditure, which means that we shall determine revenue according to expenditures, reach a general balance, and have a slight surplus. When expenditures exceed revenues, the government should draw on the previous balance of the fund, increase financial subsidies, further expand coverage, or properly increase the insurance rate through the adjustment mechanism, which should be within the acceptable range of society. Therefore, if the fund balance from the previous year is relatively low, the government will increase its financial subsidy for basic medical insurance for urban and rural residents; if the fund balance is relatively high, the government will keep its financial subsidy or provide a slightly increased amount.

**Fig. 1:**
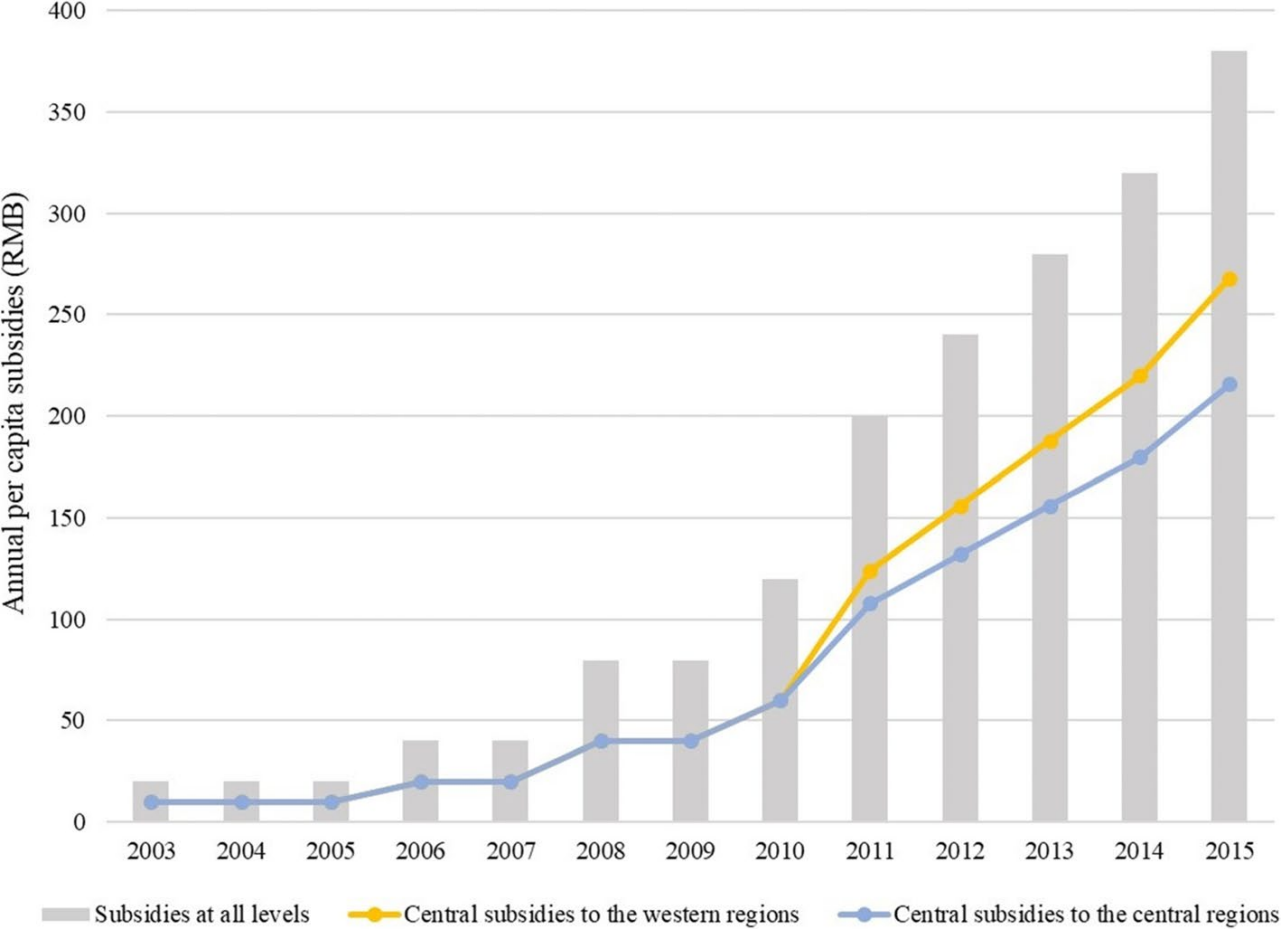
Government subsidies for NCMS. Data source: NCMS financing policy documents over the years. The detailed standards of central and local subsidies since 2010 are calculated from the related policy documents. Note that the standard of the central government’s subsidy to the eastern region and that of the local governments in this region are not provided because there is only the stipulation of “providing a subsidy according to a certain proportion” in financing documents, but no explicit stipulation concerning the central government’s subsidy to the farmer participants in the eastern regions over the year

In general, the central government takes greater responsibility for providing financial assistance. As indicated in Fig. 1, before 2011, the central government and local governments in the western region equally shared the responsibility of subsidizing health care. Since 2011, the central government has given considerable financial support to the central and western regions in terms of NCMS, especially to the western region. From, 2010 to 2018, its per capita financial subsidy to the western region increased from RMB60 to RMB356, while in the same period, the increase for the central regions was from RMB60 to RMB282.

The central government’s financial subsidy is allocated to local governments by transfer payments. The appropriation of the central financial fund follows the convention of “settling accounts in the subsequent year according to the fact”, namely, that the subsidy fund of the previous year is settled based on the actual number of participants and the standard central subsidies by the end of June in that fiscal year. For example, when settling the financial subsidy to Hubei Province in June 2013, the central government synthetically considered multiple factors, including the confirmed number of participants, the standard central subsidy, the preallocated central subsidy, and the deduction^6^ of funds due to imperfect coordination by the local government. Among these, the deduction can be treated as the central government’s punishment of local governments that fail to provide the matching subsidy.

## Methods

### Theoretical explanation

By drawing on the analysis of Ma [14], we constructed a dynamic central-local game model and then investigated the influence of central transfer payments on the behaviors of local governments, as well as their influence on the expenditure of NCMS fund.

It is assumed that the objective function of the central government is the equalized capacity of providing public service (NCMS) between regions, while that of the local government is to realize the maximum net income from the NCMS fund. The NCMS fund revenues are related to not only the central subsidy, local matching subsidy, and individual contribution but also the efforts of local governments to collect and manage the NCMS fund. Moreover, the transfer payments to be obtained by local governments in different regions also involve the regional economic development level and matching subsidies from local governments. Since the local government can restrain the behavior of its subordinates through various mechanism designs [15], we do not consider the principal-agent problem within subordinate governments; that is, we regard the local government and its affiliates as a whole. We assume that local governments take the initiative to provide matching subsidies. The reason is as follows. As mentioned above, when the central government provides financial subsidies to participants of farmers, local governments shall provide matching subsidies; when local governments fail to provide matching subsidies in time or the subsidies provided by local governments are not in full, the central government will decrease its financial subsidies by a certain ratio, and in the future, local governments will have to make up the funds deducted by the central government, in addition to the matching subsidies. Therefore, it is not worth it for local governments not to provide matching subsidies in a timely or adequate manner.

Here, we assume that there is one central government and two local governments. The game between central and local governments is a Stackelberg game, and their behaviors follow a sequential order, with one party following the other. The game between the two local governments is a Cournot game, and the sequence of their behaviors does not influence the result of their game.

All parties know that the central government will publish per capital funding and subsidy standards annually, and allocate the responsibility for financial subsidies between the central government and local governments. Thus, we assume that the government subsidy standard per capita in a certain year is S, and ${x}_{i}$($0\le {x}_{i}\le 1$) denotes the government subsidy that the central government shares with the local government i (i = 1, 2), also known as the central government sharing coefficient in this paper. Therefore, the central-to-local subsidy standard per capita can be expressed by ${\mathrm{x}}_{\mathrm{i}}\mathrm{S}$. The central government pursues the equalization of public services, which means that the smaller the gap in the capability of providing basic public services between local governments 1 and 2, the better. In mathematical form, $\left|\left({x}_{1}S+{y}_{1}\right)-\left({x}_{2}S+{y}_{2}\right)\right|$ should be as small as possible. As we all know, the smaller the absolute value of the difference is, the larger the product is. Thus, the absolute minimum of the difference can be converted to the maximum of the product, that is $\left[\left({x}_{1}S+{y}_{1}\right)\times \left({x}_{2}S+{y}_{2}\right)\right]$ should be as large as possible. For ease of calculation, after the transformation with the logarithmic function, the objective function of the central government can be expressed by Eq. 1:

1 $$\underset{{x}_{1},{x}_{2}}{\mathrm{max}}{U}_{C}=\mathrm{ln}\left({x}_{1}S+{y}_{1}\right)+\mathrm{ln}\left({x}_{2}S+{y}_{2}\right)$$

$$\mathrm{s}.\mathrm{t}. {m}_{1}{x}_{1}S+{m}_{2}{x}_{2}S\le R$$

where ${x}_{1} and {x}_{2}$ represent the subsidy sharing coefficients of the central government to local governments 1 and 2, respectively; $\left({x}_{1}S+{y}_{1}\right)$ denotes the capability of government 1 in providing an NCMS subsidy, which is constructed of the subsidy per capita from the central government ${x}_{1}S$ and the matching subsidy per capita of the local government ${y}_{1}$; $\left({x}_{2}S+{y}_{2}\right)$ denotes the capability of government 2 in providing an NCMS subsidy, which is also constructed of the subsidy per capita from the central government ${x}_{2}S$ and the matching subsidy per capita of the local government ${y}_{2}$; ${m}_{1}$ and ${m}_{2}$ represent the numbers of participants in the two local regions; and the constraint ${m}_{1}{x}_{1}S+{m}_{2}{x}_{2}S\le R$ indicates that the sum of the central subsidies to local governments 1 and 2, ${m}_{1}{x}_{1}S$ and ${m}_{2}{x}_{2}S$, respectively, shall not be higher than the central revenue.^7^ Government subsidies to NCMS are influenced by multiple factors, among which two are crucial, namely, the number of participants and the government subsidy standard.

The financial subsidy to be received by the local government $i$ is influenced by the subsidy standard S and the sharing coefficient ${x}_{i}$. The central government’s adjustment of these two indexes will affect the subsidy obtained by the local government *i*. It is given that the NCMS fund is constituted by government subsidies and individual contributions. The standard for individual contributions is assumed to be ${z}_{i}$. For the convenience of analysis, ${z}_{i}={\beta }_{i}{y}_{i}$ means that the individual contribution standard is a function of the local matching subsidy. ${\beta }_{i}$ can be interpreted as a parameter of the economic development level. The higher the level is, the larger the value of ${\beta }_{i}$ is. Here, ${0<\beta }_{i}\le 1$. Although the central government issues the corresponding annual document to stipulate the individual contribution standard of the year, that standard is generally instructive. In the application of the policy, the local government will set the local NCMS individual contribution standard according to the economic development level and affordability for farmers in the local area. Therefore, we assume the individual contribution standard to be a function of the local government’s subsidy standard.

Local governments’ efforts in fundraising and supervision will also affect the NCMS fund. More effort in fundraising and supervision means greater revenues and fewer unreasonable expenditures caused by the illegal withdrawal of medical insurance fund. However, fund-raising and supervision also incur costs, and more such efforts result in higher costs. Moreover, the marginal cost of fundraising and supervision increases gradually. The objective function of local governments can be expressed by Eq. 2:

2 $$\underset{{y}_{i}}{\mathrm{max}}{U}_{L}={m}_{i}\left({x}_{i}S+{y}_{i}+{\beta }_{i}{y}_{i}-S\right)-C$$

Local government *i* chooses to provide matching subsidy ${y}_{i}$ for maximum net income, which is influenced by both fund revenue and effort of collection and management. As in ${m}_{i}\left({x}_{i}S+{y}_{i}+{\beta }_{i}{y}_{i}\right)$, the total income of the NCMS fund is formed by government subsidy ${m}_{i}$ (${x}_{i}S+{y}_{i}$) and individual contribution ${m}_{i}$ (${\beta }_{i}{y}_{i}$). $\left({x}_{i}S+{y}_{i}+{\beta }_{i}{y}_{i}-S\right)$ indicates the extra part in the sum of the central financial subsidy and local matching subsidy compared with the government subsidy standard, namely, ${x}_{i}S+{y}_{i}\ge S$, or ${x}_{i}S+{y}_{i}-S\ge 0$. C is the cost of collection and management, which is related to fund revenue. For the convenience of analysis, $\mathrm{C}={\alpha }_{i}{\left[{m}_{i}\left({x}_{i}S+{y}_{i}+{\beta }_{i}{y}_{i}\right)\right]}^{2}$ is the cost of collection and management as a function of fund revenue. The quadratic component in this equation indicates the marginal increasing characteristic of C. ${\alpha }_{i}$ is a parameter of the economic development level. A high economic development level contributes to the innovation of collection and management technology and measures. Therefore, the higher the economic development level is, the smaller the value of ${\alpha }_{i}$ is. Here, ${\alpha }_{i}>0$. Then, Eq. 2 can be transformed into Eq. 3:

3 $$\underset{{y}_{i}}{\mathrm{max}}{U}_{L}={m}_{i}\left({x}_{i}S+{y}_{i}+{\beta }_{i}{y}_{i}-S\right)-{\alpha }_{i}{\left[{m}_{i}\left({x}_{i}S+{y}_{i}+{\beta }_{i}{y}_{i}\right)\right]}^{2}$$

Previous studies have demonstrated that financial strength affects local governments’ expectations of receiving payment transfers. Local governments in strong financial positions anticipate smaller subsidies; thus, they are motivated to take measures to control financial expenditures [10, 16]. Therefore, this study investigates the behaviors of local governments under the two situations of stronger and weaker financial positions. Local governments in strong financial positions move first, and the central government moves accordingly, while local governments in weaker financial positions take their cue from the central government’s first move.

#### With a weaker local financial strength

In the case of local governments with relatively weak financial positions, the central government moves first, and the local governments follow accordingly. There is a dynamic game between the central and local governments and a static game between local governments 1 and 2. First, the central government determines its sharing coefficients ${x}_{1}$ and ${x}_{2}$, while according to the observed central subsidy standards, local governments 1 and 2 determine their matching subsidy levels ${y}_{1}$ and ${y}_{2}$. The behaviors of local governments are first considered. Due to the static game between local governments 1 and 2, the order of analysis between the two does not impact the results of the game. Here, the behaviors of local government 1 are first considered. When (*x*_1_, *x*_2_) and ${y}_{2}$ are given, local government 1 chooses ${y}_{1}$ to realize its objective of maximum net income. Then, Eq. 3 becomes:

4 $$\underset{{y}_{1}}{\mathrm{max}}{U}_{L}={m}_{1}\left({x}_{1}S+{y}_{1}+{\beta }_{1}{y}_{1}-S\right)-{\alpha }_{1}{\left[{m}_{1}\left({x}_{1}S+{y}_{1}+{\beta }_{1}{y}_{1}\right)\right]}^{2}$$

Solve the optimization problem of Eq. 4, and the response function of local governments 1 and 2 can be obtained as

5 $${y}_{1}^{1}=\frac{1}{2\left(1+{\beta }_{1}\right){\alpha }_{1}{m}_{1}}-\frac{S}{1+{\beta }_{1}}{x}_{1}$$

6 $${y}_{2}^{1}=\frac{1}{2\left(1+{\beta }_{2}\right){\alpha }_{2}{m}_{2}}-\frac{S}{1+{\beta }_{2}}{x}_{2}$$

Equations 5 and 6 indicate that there is a negative correlation between the local matching subsidy and the central subsidy, and the level of the latter will influence the initiative of the former. Similarly, the local matching subsidy has a positive correlation with the number of local participants. With more participants, the local matching subsidy standard will be lower. In addition, the local matching subsidy is a decreasing function of the supervision cost coefficient. With a larger supervision cost coefficient, supervision will be more difficult, and the local matching subsidy will be lower.

Then, the behaviors of the central government are analyzed. The response functions of local governments 1 and 2, which are known by the central government, act as constraint conditions on the central government. Then, the objective function of the central government becomes

$$\underset{{x}_{1}, {x}_{2}}{\mathrm{max}}{U}_{C}=\mathrm{ln}\left({x}_{1}S+{y}_{1}^{1}\right)+\mathrm{ln}\left({x}_{2}S+{y}_{2}^{1}\right)$$

$$\mathrm{s}.\mathrm{t}. {m}_{1}{x}_{1}S+{m}_{2}{x}_{2}S\le R$$

By substituting Eq. (5) and (6) and constructing a Lagrange function, we obtain

$$\mathrm{L}=\mathrm{ln}\left[\frac{1}{2\left(1+{\beta }_{1}\right){\alpha }_{1}{m}_{1}}+\frac{{\beta }_{1}S}{1+{\beta }_{1}}{x}_{1}\right]+\mathrm{ln}\left[\frac{1}{2\left(1+{\beta }_{2}\right){\alpha }_{2}{m}_{2}}+\frac{{\beta }_{2}S}{1+{\beta }_{2}}{x}_{2}\right]-\uplambda \left({m}_{1}{x}_{1}S+{m}_{2}{x}_{2}S-R\right)$$

The first-order conditions of the optimization problem are

$$\frac{\partial L}{\partial {x}_{1}}=\frac{2{\alpha }_{1}{\beta }_{1}{m}_{1}S}{1+2{\alpha }_{1}{\beta }_{1}{m}_{1}{x}_{1}S}-\lambda {m}_{1}S=0$$

$$\frac{\partial L}{\partial {x}_{2}}=\frac{2{\alpha }_{2}{\beta }_{2}{m}_{2}S}{1+2{\alpha }_{2}{\beta }_{2}{m}_{2}{x}_{2}S}-\lambda {m}_{2}S=0$$

$$\frac{\partial L}{\partial \lambda }={m}_{1}{x}_{1}S+{m}_{2}{x}_{2}S-R=0$$

By solving this equation, we obtain

7 $${x}_{1}^{1}=\frac{{\alpha }_{1}{\beta }_{1}+ZR-{\alpha }_{2}{\beta }_{2}}{2{m}_{1}ZS}$$

8 $${x}_{2}^{1}=\frac{{\alpha }_{2}{\beta }_{2}+ZR-{\alpha }_{1}{\beta }_{1}}{2{m}_{2}ZS}$$

Equations 7 and 8 denote determinations of the central government, where $\mathrm{Z}=2{\alpha }_{1}{\beta }_{1}{\alpha }_{2}{\beta }_{2}$. They indicate that the central sharing coefficient has a positive correlation with the central government’s revenues. With larger revenues, the sharing coefficient will be correspondingly larger. The central sharing coefficient has a negative correlation with the number of local participants and the government subsidy standard.

#### With a stronger local financial strength

Local governments with stronger financial positions determine their matching subsidies first, and then the central government adjusts its subsidies according to the matching subsidies it observes. Here, we first investigate the behaviors of the central government. Given local matching subsidies ${y}_{1}$ and ${y}_{2}$, the central government determines its sharing coefficients ${x}_{1}\mathrm{and }{x}_{2}$, thus realizing the equalized capability of providing NCMS between the two. Then, the response equation of the central governments can be obtained as

9 $${x}_{1}\left({y}_{1},{y}_{2}\right)=\frac{{m}_{2}{y}_{2}+R-{m}_{1}{y}_{1}}{2{m}_{1}S}$$

10 $${x}_{2}\left({y}_{1},{y}_{2}\right)=\frac{{m}_{1}{y}_{1}+R-{m}_{2}{y}_{2}}{2{m}_{2}S}$$

The above equations denote that the central sharing coefficients are functions of the local matching subsidies ${y}_{1}$ and ${y}_{2}$ and are positively related to the central financial revenue and negatively related to the number of local participants. The central government’s financial subsidy sharing coefficient for either local government is positively correlated with the latter’s matching subsidy but negatively correlated with the matching subsidy of the other. This further proves that the central government pursues fairness and emphasizes equity in NCMS funding across regions.

Then, the behaviors of the local governments are analyzed. The response function of the central government acts as a constraint of the local government, which pursues maximization of utility:

$$\underset{{y}_{i}}{\mathrm{max}}{U}_{L}={m}_{i}\left({x}_{i}S+{y}_{i}+{\beta }_{i}{y}_{i}-S\right)-{\alpha }_{i}{\left[{m}_{i}\left({x}_{i}S+{y}_{i}+{\beta }_{i}{y}_{i}\right)\right]}^{2}$$

$$\mathrm{s}.\mathrm{t}. {x}_{1}\left({y}_{1},{y}_{2}\right)=\frac{{m}_{2}{y}_{2}+R-{m}_{1}{y}_{1}}{2{m}_{1}S}$$

$${x}_{2}\left({y}_{1},{y}_{2}\right)=\frac{{m}_{1}{y}_{1}+R-{m}_{2}{y}_{2}}{2{m}_{2}S}$$

Solving the above, we can obtain

11 $${y}_{1}=\frac{1-{\alpha }_{1}\left({m}_{2}{y}_{2}+R\right)}{{\alpha }_{1}{m}_{1}\left(1+2{\beta }_{1}\right)}$$

12 $${y}_{2}=\frac{1-{\alpha }_{2}\left({m}_{1}{y}_{1}+R\right)}{{\alpha }_{2}{m}_{2}\left(1+2{\beta }_{2}\right)}$$

Equations 11 and 12 represent the matching subsidy standards determined by the local governments. They indicate that the matching subsidy standard of local government 1 is negatively correlated with that of local government 2, the central government’s revenues and the number of local participants. Given a local government’s NCMS subsidy, an increasing number of participants indicates a decreasing per capita subsidy, namely, that the local matching subsidy standard will also decrease.

In the two situations of stronger local financial position and weaker local financial position, the difference in the collection and management efforts of the local governments was analyzed. It is known that the local government pursues its maximum net income. Thus, the smaller collection and management costs are, the better chance of achieving that maximum net income. Then, we need to know in which situation the local governments’ collection and management costs can be lower, namely, a lower value of ${\alpha }_{i}{\left[{m}_{i}\left({x}_{i}S+{y}_{i}+{\beta }_{i}{y}_{i}\right)\right]}^{2}$. Thus, taking local government 1 as an example, this question can be solved by exploring whether ${\alpha }_{1}{\left[{m}_{1}\left({x}_{1}S+{y}_{1}+{\beta }_{1}{y}_{1}\right)\right]}^{2}<{\alpha }_{1}{\left[{m}_{1}\left({x}_{1}^{1}S+{y}_{1}^{1}+{\beta }_{1}{y}_{1}^{1}\right)\right]}^{2}$ is true.

13 $$\frac{{\alpha }_{1}{\left[{m}_{1}\left({x}_{1}S+{y}_{1}+{\beta }_{1}{y}_{1}\right)\right]}^{2}}{{\alpha }_{1}{\left[{m}_{1}\left({x}_{1}^{1}S+{y}_{1}^{1}+{\beta }_{1}{y}_{1}^{1}\right)\right]}^{2}}<1$$

Substitute ${x}_{1}$,${y}_{1}$,${x}_{1}^{1}\mathrm{ and }{y}_{1}^{1}$ into Eq. 13, and we can obtain through calculation that when $\left({m}_{2}{y}_{2}+R+2{\beta }_{1}{m}_{1}{y}_{1}+{m}_{1}{y}_{1}\right)<\frac{1}{{\alpha }_{1}}$, Eq. 13 is true. 8 That is, the collection and management costs are lower for local governments with stronger financial strength. Local governments with a weaker financial position may engage in strategic behavior that aims to acquire greater subsidies from the central government by reducing its collection and management efforts and costs.

### Data

Panel data from 28 provinces in mainland China from 2004 to 2014 were collected for this study. We use 2004 as the initial year because it is the year after the NCMS was established. We use 2014 as the final year because it is the last year available in the data.^9^ Tianjin, Shandong, and Guangdong were excluded due to the serious lack of NCMS revenue and expenditure data. The data were from the “China Statistical Yearbook”, “China Health and Family Planning Statistical Yearbook”, “Finance Yearbook of China”, and “New Rural Cooperative Medical System Statistics”.

The number of NCMS participants, central subsidy (transfer payment), local (matching) subsidy, and revenue and expenditure data come from the “New Rural Cooperative Medical System Statistics”. Part of the missing data are complemented with data from the “China Health and Family Planning Statistical Yearbook”. The local economic development level, level of aging (proportion of the population aged 65 and over), and proportion of the population below 14 years old are mainly from the “China Statistical Yearbook”. The indicators for constructing fiscal decentralization are cited from the “Finance Yearbook of China”. To remove the influence of price and inflation, we take 2004 as the benchmark year, taking the price of this year as the base price, to deflate the income and expenditure data. Additionally, to remove the influence of outliers, we conducted a logarithmic transformation of the data after deflation. Logarithmic transformation also makes different variables comparable.^10^

### Statistical analysis

Referring to the studies of Pettersson-Lidbom and Dahlberg [17], Pettersson-Lidbom [5] and Wang [2], this study mainly inspects two models in the empirical procedure: the transfer payment model and the fund balance model. With the transfer payment model, we investigate the factors influencing central transfer payments and study the impact of transfer payments on the NCMS fund balance by taking the fitted value of transfer payments as an explanatory variable. Model 1 and Model 2 are reference models.^11^ The Hausman test [18] was used to determine the appropriateness of the random effect model and the fixed effect model.

Model 1 $${expect}_{it}={\alpha }_{1}{participation}_{it}+{\alpha }_{2}{GDP}_{it}+{\alpha }_{3}{balance}_{it-1}+{\alpha }_{4}{cfinc}_{t}+region+{\mu }_{i}+{\lambda }_{t}+{\varepsilon }_{it}$$

Model 2 $${balance}_{it}={\beta }_{1}{\widehat{expect}}_{it}+{\beta }_{2}l{transfer}_{it}+\delta {x}_{it}+{\mu }_{i}+{\lambda }_{t}+{\varepsilon }_{it}$$

Considering the interaction of NCMS expenditure decisions between different provinces, this paper, based on the reference models, brings in the transfer payment obtained by the neighboring province in the previous year as the instrumental variable of the transfer payment obtained by the current region under research. Then, with the dynamic panel model, the one-step system GMM method is adopted for regression, as in Models 3 and 4.

Model 3 $${expect}_{it}=\alpha {ltransfer}_{it-1}+\varphi {\sum }_{W}{transfer}_{it-1}+\delta {x}_{it}+{\mu }_{i}+{\lambda }_{t}+{\varepsilon }_{it}$$

Model 4 $${balance}_{it}={\beta }_{0}+{\beta }_{1}{balance}_{it-1}+{\beta }_{2}{\widehat{expect}}_{it}+\delta {x}_{it}+{\mu }_{i}+{\lambda }_{t}+{\varepsilon }_{it}$$

**Fig. 2:**
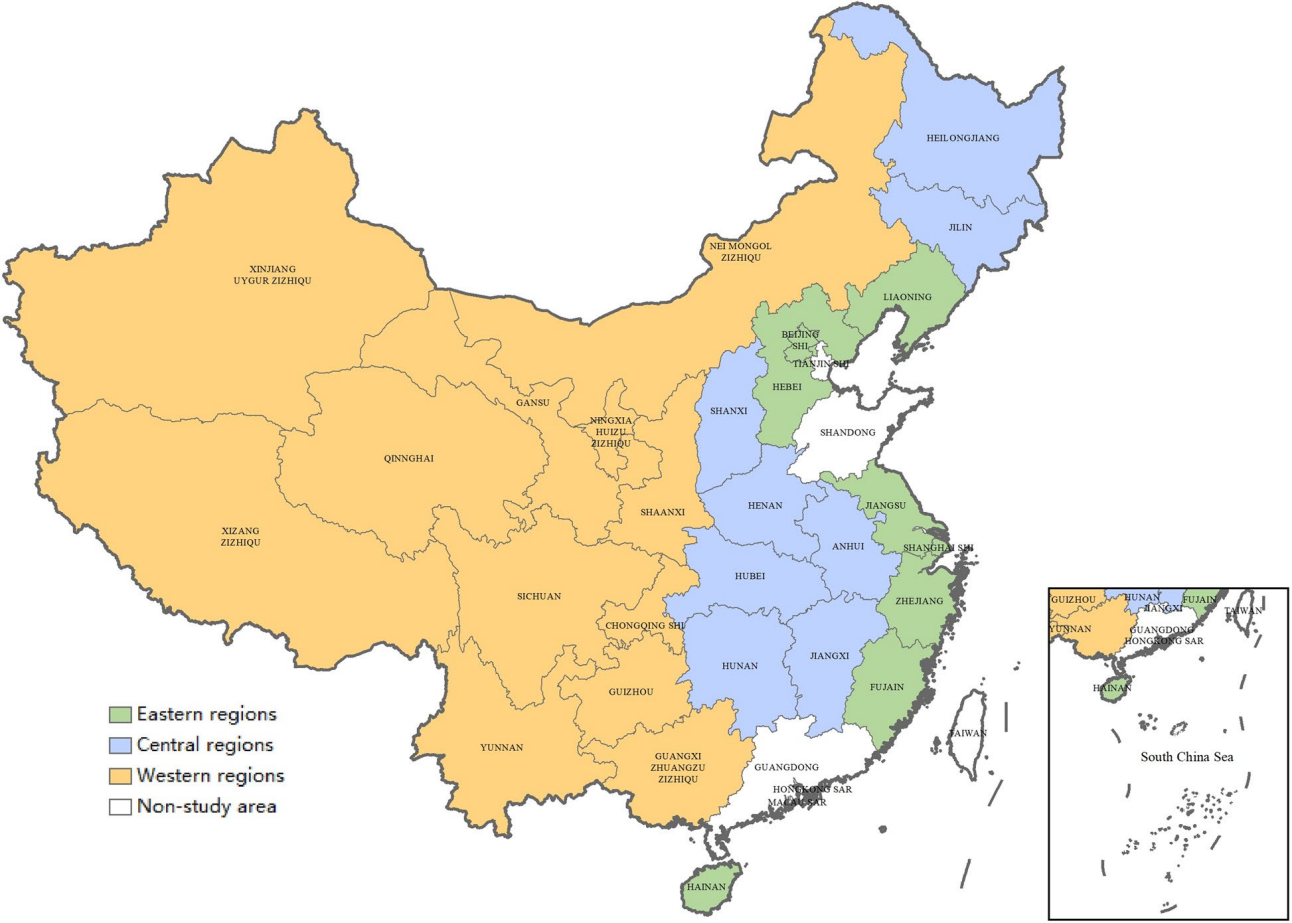
The distribution of three regions in China. In this study, the eastern regions include 8 provinces (autonomous regions and municipalities), including Beijing, Hebei, Liaoning, Shanghai, Jiangsu, Zhejiang, Fujian, and Hainan; the central regions encompass 8 provinces, including Shanxi, Jilin, Heilongjiang, Anhui, Jiangxi, Henan, Hubei, and Hunan; the western regions include 12 provinces (autonomous regions and municipalities), including Nei Mongol, Guangxi, Chongqing, Sichuan, Guizhou, Yunnan, Xizang, Shaanxi, Gansu, Qinghai, Ningxia, and Xinjiang

${expect}_{it}$ refers to the expected central transfer payment that the local government *i* can obtain in the term *t*;${balance}_{it}$ is the current balance rate of NCMS fund, which is represented by the ratio of NCMS fund revenue-expenditure gap and the revenue in the current period;${balance}_{it-1}$ reflects the balance rate of the region *i* in the *t-1* term; ${\sum }_{W}{transfer}_{it-1}$ is the spatial weighted variable of transfer payment, namely, the transfer payment that the neighboring province obtained in the previous term; ${\mathrm{ltransfer}}_{\mathrm{it}}$ indicates the local matching subsidy for NCMS fund (as mentioned above, the government subsidy for NCMS fund is constituted by the central subsidy and local matching subsidy, and with a given central transfer payment and fund expenditure, the NCMS balance increases with the local matching subsidy); ${participation}_{it}$ represents the number of NCMS participants in the region *i* (in logarithmic form);${GDP}_{it}$ reflects the economic development level in this region (per capita GDP, in logarithmic form); ${cfinc}_{t}$ indicates the financial revenues of the central government in term *t* (in logarithmic form); $region$ represents the regional factor, which is 0 for eastern regions, 1 for central regions, and 2 for western regions, the distribution of those regions is shown in Fig. 2; and ${\mathrm{x}}_{\mathrm{it}}$ indicates a series of control variables,${\mu }_{i}$ is the provincial fixed effect, ${\lambda }_{t}$ is the time fixed effect, and ${\varepsilon }_{it}$ is a random disturbance term. Referring to Zhu et al. [19] and Wang et al. [20], we choose old (degree of aging), children (proportion of the population below 14 years old), healthcare-exp (per capita health care expenditures, in logarithmic form), and last-term balance rate of NCMS fund as the control variables.

Spatial correlation is a precondition for establishing a spatial econometric model. The indexes of Moran’s l and Geary’s C, etc., are generally adopted for the inspection of spatial correlation, but these are mainly for cross-sectional data and “cannot be directly applied in the panel data model” [21]. Hence, by referring to the methods of Wang and Shen [21] and Li et al. [22], this study brings Moran’s l, etc., into the panel data model by constructing the block matrix. Concerning the setting of the spatial weight matrix, three construction methods are generally adopted in the existing research, namely, the geological space, economic development level, and all other regions [21, 22]. For the convenience of research, based on the data of 28 provinces, cities and arch autonomous regions, this study constructs a spatial weight matrix according to the geological space, taking the neighboring province as 1 and the nonadjacent province as 0.

## Results

### Description

The variables are described in Table 1. We used 308 province/year observation samples. It can be seen that the fund balance rate varies greatly among provinces (the smallest is only -17.306%, and the largest province is 100%), and so does the fund balance rate in the first-stage lag (the smallest is only -12.629%, and the largest province is 100%, which was in 2004). In addition, the central government's transfer payments to provincial medical insurance fund vary greatly (the standard deviation is 3.359), and there is also a significant difference between local matching subsidies (the standard deviation is 1.768). The average proportion of people over 65 years old is 8.963%, but there is a large difference among samples; the minimum value appeared in 2011 in Xizang (4.824%), and the maximum value appeared in 2014 in Shanghai (15.339%). Similarly, the minimum proportion of children under 14 years old appeared in 2014 in Shanghai (7.559%), and the maximum value appeared in 2004 in Guizhou (28.336%). The difference in per capita medical expenditures in the sample (standard deviation is 1.064) far exceeds the per capita GDP (standard deviation is 0.575) and the central government revenues (standard deviation is 0.402).

**Table 1:** Descriptive statistics of the main variables

Variables	Mean	Std. Dev	Min	Max
Transfer (log)	9.720	3.359	0.000	13.999
Participation (log)	7.008	1.289	3.490	9.019
Per capita GDP (log)	9.869	0.575	8.366	11.337
Region	1.143	0.834	0.000	2.000
Cfinc (log)	7.585	0.402	6.924	8.323
Lagged one period of balance rate	16.565	17.083	-12.629	100.000
Balance rate	15.380	16.874	-17.306	100.000
Local matching subsidy(log)	10.795	1.768	5.947	15.906
Old	8.963	1.811	4.824	15.399
Children	17.731	4.434	7.559	28.336
Healthcare-exp (log)	4.470	1.064	0.000	7.399

*Std. Dev* Standard deviation, *Min* Minimum, *Max* Maximum

### Pooled OLS regression

In the empirical analysis, this paper takes the panel data as cross-sectional data and carries out pooled OLS regression. Then, considering heterogeneity between provinces, the fixed-effect model and random-effect model are adapted for regression. The Hausman test result shows that the transfer payment model (reference model) should select a random effect model (*p* = 0.1245), and the expenditure model of the NCMS fund (reference model) should choose a fixed effect model (*p* = 0.0000). The regression analysis results are shown in Tables 2 and 3.

**Table 2:** Transfer payment model (reference model)

	**(1)** **Pooled OLS**	**(2)** **Fixed effect**	**(3)** **Random effect**
Participation (log)	1.205^***^	0.718***	0.953***
	(8.766)	(3.145)	(7.453)
Per capita GDP (log)	-3.052^***^	-2.162**	-3.040***
	(-3.291)	(-2.135)	(-3.120)
Region	0.413	0.000	0.432
	(1.139)	(.)	(1.510)
Cfinc (log)	5.562^***^	5.265***	5.753***
	(6.702)	(4.795)	(6.046)
Lagged one period of balance rate	-0.009^*^	-0.016**	-0.015***
	(-1.903)	(-2.762)	(-2.933)
Constant	-11.152^**^	-13.624***	-10.831***
	(-2.691)	(-4.396)	(-3.021)
Observations	280	280	280
R^2^	0.773	0.760	0.753

*, **, and *** indicate significance at the levels of 10%, 5%, and 1%, respectively

**Table 3:** Expenditure model of NCMS fund (reference model)

	**(1)** **Pooled OLS**	**(2)** **Fixed effect**	**(3)** **Two-way fixed effect**	**(4)** **Random effect**
Fitted value of the transfer	-0.035	-1.866^**^	-3.656*	0.225
	(-0.033)	(-2.082)	(-1.920)	(0.332)
Local matching subsidy (log)	-0.773	6.341^***^	0.355	0.085
	(-0.440)	(4.895)	(0.134)	(0.075)
Per capita GDP (log)	9.731	71.037^***^	3.420	15.762^***^
	(1.551)	(10.852)	(0.387)	(3.303)
Old	0.313	-1.002	1.609***	-0.030
	(0.580)	(-1.574)	(3.280)	(-0.058)
Children	0.721^**^	0.680	0.928**	0.905^***^
	(2.218)	(1.366)	(2.340)	(2.706)
Healthcare-exp (log)	-11.226^***^	-39.189^***^	-22.963***	-14.784^***^
	(-4.271)	(-15.863)	(-4.934)	(-8.085)
Province fixed effect	NO	YES	YES	NO
Time fixed effect	NO	NO	YES	NO
Constant	-38.537	-565.776^***^	50.611	-94.253^**^
	(-0.720)	(-10.086)	(0.599)	(-2.180)
Observations	280	280	280	280
R^2^	0.450	0.653	0.745	0.535

*, **, and *** indicate significance at the levels of 10%, 5%, and 1%, respectively

As shown in column 3 of Table 2, the number of NCMS participants has a significant positive influence on the transfer payment to be obtained, with a coefficient of 0.953 (*p* < 0.01), which indicates that a larger number means a higher transfer payment. This agrees with the fact that with a fixed per capita subsidy standard, the central government will provide more financial subsidies to the region with more NCMS participants. The influence of per capita GDP on the transfer payment to be obtained is significantly negative, with a coefficient of -3.040 (*p* < 0.01). In regions with a higher GDP, the transfer payments will be lower. The central government’s financial revenues have a significant positive impact on the transfer payments to be obtained (5.753, *p* < 0.01), which agrees with the theoretical analysis. According to the theoretical analysis, the central financial strength and the sharing coefficient of central transfer payments have a positive correlation, meaning that with stronger central financial strength, the central sharing coefficient will be higher, since the central government will have more capital for transfer payments. The impact of a one-period lag of the balance rate on transfer payments is significantly negative, while the coefficient is very small (-0.015).

As shown in column 3 of Table 3, the fitted value of transfer payments has a significant negative impact on the NCMS fund balance rate, with a coefficient of -3.656 (*p* < 0.10). The per capita GDP is positively correlated with the fund balance, but it is not significant. The matching subsidies of local governments have a positive effect on the fund balance rate, but the effect is not significant. The proportion of the population aged 65 and older and the population aged less than 14 have a significant impact on the fund balance rate. The per capita health care expenditure and the NCMS fund balance rate have a significant negative correlation.

### IV analysis

Using the fitted value of transfer payments as the explanatory variable may cause problems due to missing variables and endogeneity. Referring to the research of Liu et al. [23] and Xie and He [24], we take the last-term transfer payment obtained by the neighboring province as the instrumental variable to investigate the influence of central transfer payments on NCMS fund expenditures.

Taking 2010 as an example, through the construction of the block matrix, the inspection result shows that Moran’s l is equal to 0.509 (*p* = 0.000), which strongly rejects the original hypothesis of “no spatial autocorrelation”, namely, admitting the existence of spatial correlation.

Table 4 reports the estimated results of models 3. The first column is the result of the Static Space Panel. Column 2–4 is the results of the dynamic space panel, including provincial fixed effect, time fixed effect and both. Hausman test result shows that the random effect model (*p* = -6.68) should be selected for static space panel. Considering various factors, the model with provincial fixed effect is selected in the dynamic space panel.

**Table 4:** Transfer payment model

	**(1)** **Static panel model**	**(2)** **Dynamic panel model (fixed effect)**	**(3)** **Dynamic panel model (time fixed effect)**	**(4)** **Dynamic spatial panel model (both)**
Participation (log)	1.185***	0.416**	0.365***	0.226
	(9.621)	(1.998)	(4.662)	(1.042)
Lagged one period of transfer		0.473***	0.753***	0.468***
		(9.771)	(18.281)	(9.884)
Lagged one period of neighbor transfer		-0.299***	-0.084	-0.171
		(-3.561)	(-0.952)	(-1.624)
Per capita GDP (log)	-2.888***	-1.396*	-0.793***	-1.705
	(-6.261)	(-1.956)	(-3.015)	(-1.507)
Region	0.302	0.000	-0.069	0.000
	(1.101)	(.)	(-0.573)	(.)
Cfinc	5.113***	2.519***	0.444	3.224*
	(9.200)	(2.975)	(0.439)	(1.903)
Lagged one period of balance rate	-0.011**	-0.009*	0.006	-0.003
	(-2.297)	(-1.860)	(1.119)	(-0.634)
Neighbor participation	-0.553***	0.036	-0.247*	-0.751**
	(-3.314)	(0.152)	(-1.763)	(-2.193)
Spatial correlation	0.255***	0.345***	0.008	0.013
	(3.806)	(4.952)	(0.091)	(0.146)
Log-likelihood	-518.408	-391.078	-460.144	-518.408
R^2^	0.791	0.849	0.838	0.792
AIC	20.000	18.000	20.000	18.000
BIC	57.301	50.713	56.348	50.713
Observations	308	280	280	280

*, **, and *** indicate significance at the levels of 10%, 5%, and 1%, respectively. The region is a time-invariant variable

As shown in column 2 of Table 4, the analysis result of the dynamic space panel shows that the last-term central transfer payment to a certain region has a significant positive influence on the transfer payment obtained in the current term, with a coefficient of 0.473 (*p* < 0.05). The per capita GDP has a negative influence on the transfer payments obtained by a region, which is significant at 90% confidence intervals. The central fiscal revenue is positively correlated with the transfer payments received by local governments, with a coefficient of 2.519 (*p* < 0.05). The last-term balance rate of the NCMS fund has a negative influence on the transfer payment that the local government expects to obtain, while the coefficient is very small (-0.009, *p* < 0.1). These results are consistent with the theoretical analysis.

However, the last-term central transfer payment obtained by the neighboring province has a negative correlation with the transfer payment to be obtained by the current region in this term, with significance at the 95% confidence interval. The participation of the neighboring province has no significant impact on the transfer payment.

Table 5 reports the estimation results of Model 4. In Table 5, the estimation results after adding the indicators of financial revenue decentralization and financial expenditure decentralization are described. Among these, the fixed effect models 1 and 2^12^are the estimation results of static panels, while the one-step GMM models 1 and 2 are the estimation results of dynamic panels. To address the potentially missing variables and endogeneity, this study closely considers the regression result of the one-step GMM estimation model.

**Table 5:** NCMS fund expenditure model

	(1) **Fixed model 1**	(2) **Fixed model 2**	(3) **One step GMM 1**	(4) **One step GMM 2**
Transfer	-3.291***	-4.096***	-2.380**	-1.334
	(0.601)	(0.804)	(0.978)	(0.963)
Lagged one period of balance rate			-0.242***	-0.190***
			(0.071)	(0.057)
Lagged two period of balance rate			-0.248***	-0.181***
			(0.060)	(0.060)
Lagged one period of expectation			-4.323***	-3.435***
			(1.293)	(0.988)
Revenue decentralization		-0.298	-0.538	
		(0.221)	(0.442)	
Expenditure decentralization	0.396*			1.234**
	(0.226)			(0.534)
Local matching subsidy	0.921	0.070	8.637***	6.869***
	(1.058)	(1.194)	(1.837)	(1.551)
Per capita GDP	2.569	10.188*	100.332***	79.159***
	(3.907)	(5.382)	(15.666)	(11.024)
Old	1.502***	1.728***	0.727	0.980
	(0.375)	(0.444)	(0.932)	(0.882)
Children	0.748**	0.851***	2.313***	2.884***
	(0.327)	(0.300)	(0.794)	(0.722)
Healthcare expenditure	-26.964***	-25.927***	-44.234***	-44.813***
	(4.219)	(4.761)	(5.255)	(5.021)
Province fixed effect	YES	YES	NO	NO
Time fixed effect	YES	YES	NO	NO
Constant	40.418	16.001		
	(48.625)	(48.613)		
Observations	280	280	224	224
R^2^	0.7698	0.7600		
AR(1)			0.001	0.001
AR(2)			0.222	0.192
Sargan			0.000	0.000
Hansen			0.545	0.528

*, **, and *** indicate significance at the levels of 10%, 5%, and 1%, respectively. AR(1), AR(2), Sargan, and Hansen are *p* values

As shown in columns 3–4 of Table 5, the expectation of transfer payments has a significant negative impact on the balance rate of the NCMS fund (-2.38, *p* < 0.01) when financial revenue decentralization is taken into consideration. When financial expenditure decentralization is taken into consideration, such an impact is negative, but not significant. The influence of financial revenue decentralization on the balance rate of the NCMS fund is negative but not significant, while financial expenditure decentralization has a positive impact on the balance rate, with a coefficient of 1.234 (*p* < 0.01). The impact of the local matching subsidy, per capita GDP and per capita health insurance expenditure on the balance rate of NCMS agree with the analysis results listed in Table 3.

Table 5 also shows that the lagged one period of balance rate and the lagged two periods of balance rate both have negative effects on the current NCMS fund balance rate, significant at the 95% confidence intervals. Meanwhile, the lagged one period of expectation shows a significant negative impact on the current NCMS fund balance rate, with the coefficient changing from -4.323 (*P* < 0.05) to -3.435 (*P* < 0.05) when financial revenue decentralization is replaced by financial expenditure decentralization.

## Discussion

### Central transfer payments will lead to strategic behaviors among local governments, resulting in increased local health insurance fund expenditures and lower balance rates

As anticipated by theoretical analysis, the transfer payments provided by the central government for equalized capability of regions to provide basic public services result in strategic behaviors among local governments. When anticipating that the central government will provide transfer payments according to the health insurance fund balance in the previous year, local governments reduce their collection and management efforts, which lowers the NCMS fund balance and increases transfer payments from the central government. The empirical analysis results further prove that the transfer payments from central government to local governments influence the NCMS fund balance. The greater the transfer payments from the central government are, the lower the NCMS fund balance rates are. Consistent with previous studies, local governments reduce collection efforts [1] and increase financial expenditures [2–4, 25] when they receive central transfer payments.

Local governments determine their financial expenditures based on anticipation of bailouts from the central government [26]. If a local government has a high probability of transfer payments, it is likely to have higher financial expenditures. Moreover, compared with ex-ante transfer payments, ex-post transfer payments could cause excessive expenditures more easily [27]. When adding the variable of financial revenue decentralization, the expectation of transfer payments has a negative impact on the balance of NCMS fund. However, when adding the variable of financial expenditure decentralization, such an impact is not that obvious.

The central transfer payment has “path dependence”. The last-term central transfer payment to a certain region has a positive influence on the transfer payment to be obtained in the current term, which will negatively affect the balance rate of the NCMS fund. This means that the more central transfer payments a local government has received last term, the more transfer payments it expects in the current term. This, in turn, means higher NCMS fund expenditures and a lower balance rate. The central government will adjust its transfer payments according to the balance rate of the NCMS fund. With a lower balance rate in the previous year, the central government will provide a higher transfer payment in the current year. Meanwhile, if the central government provides relatively higher transfer payments to a region, that region may acquire more transfer payments in the future. This result indicated that the central transfer payment has a certain “path dependence”.

### Strengthen the responsibility of local governments and balance the medical insurance fund

The greater the local matching subsidy is, the higher the balance rate of the NCMS fund is. On the one hand, with a given NCMS fund expenditure and central subsidy, a greater local matching subsidy indicates higher fund revenues, which represents a higher fund balance under fixed fund expenditures. On the other hand, with more local matching subsidies, the local governments will have a stronger impetus to control expenditures, and there will be fewer unreasonable expenditures, resulting in a higher fund balance. This indicates that the local government should be emphasized in medical insurance fund supervision. In fact, according to relative policies of NCMS fund expenditures, it is clearly stipulated that the local government carries the responsibility of covering the deficit of funds, as well as the responsibility of supervising and decreasing unreasonable fund expenditures. However, due to the central transfer payment and the resulting strategic behavior of local governments, the responsibility of making up the fund deficit was finally transferred to the central government, and the responsibility of local governments was weakened. This suggests that the role of local governments should be given more importance to advance the quality and efficiency of medical insurance fund oversight.

### Enhance the local financial strength and balance the medical insurance fund

In the more developed regions, transfer payments are lower. The central government considers the economic development level of regions in the determination of the sharing coefficient and provides a lower share for regions at a high level of economic development and a higher share for regions at low levels of economic development. This is evidence of the central government’s pursuit of equalization through transfer payments. In regions with higher per capita GDP, the NCMS fund balance is higher. On the one hand, local governments expect lower transfer payments, which puts pressure on controlling unreasonable fund expenditures. On the other hand, these governments are able to afford more subsidies. This is concrete evidence that transfer payments lead to inefficient lock-in [8] in medical insurance.

### The obtained transfer payments of local governments have significant space correlation

The space correlation across regions is significant in transfer payments administered by the central government. The greater transfer payments that specific provinces obtained in the previous fiscal year may mean fewer transfer payments to neighboring provinces in the current fiscal year. As discussed previously, transfer payments have a “path dependence” issue. The transfer payment a region obtained before has a positive impact on the transfer payment to be obtained in the future. The more transfer payments a province has obtained, the more transfer payments it will obtain in the future. However, the total capital for central transfer payments is limited. Thus, the greater the payment to one province is, the lower the payment to the neighboring province is. The regional factor has no significant influence on the transfer payment to be obtained, and one of the possible reasons is that the regional influence has already been considered at the economic development level. As mentioned earlier, the three regions were used to represent three economic development levels in China [28–30].

The significant space correlation of the central transfer payments between provinces reflects the inequitable distribution of financial resources. The gap between the central and western regions in payment transfers has increased by as much as 10 percent in recent years. At the same time, the medical insurance fund deficit gradually appeared. It is time to establish effective incentives and restraint mechanisms, strengthen supervision mechanisms and refine the spending structure of medical insurance fund.

## Conclusions

In this paper, we find that the central transfer payment, which was designed to realize the equalized capability of providing NCMS, induced the strategic behavior of local governments, such as neglecting the supervision of medical insurance fund expenditures, thus reducing the efficiency of the management and usage of medical insurance fund. Negligence may be encouraged if strategic behavior leads to more transfer payments to more relaxed supervision. In this case, the security of medical insurance fund is impaired, and the inequality in health among different provinces could be increased. While transfer payments might help local governments balance medical insurance fund in short term, the overall cost is high. Understanding the strategic behavior of local governments is important, especially in improving the transfer payment system. Thus, if we are to achieve equity in health, we need to deal with the strategic behavior of local governments.

Medical insurance fund must be well managed and equitably distributed. We can strengthen the supervision ability and initiatives of local governments, refine the central transfer payment mechanism, promote the economic growth of western regions and localities, and appropriately increase rates for individual contributions. Other countries may encounter similar problems in the process of equalizing medical insurance resources through central transfer payments. We hope this paper provides a reference for other countries.

Via the dynamic central-local game model, this paper captures the local governments’ strategic regulatory behaviors, which were induced by transfer payments. We suppose it has caused health inequity among provinces. We leave to future research on the issue of how transfer payments affect the equity in health among individuals through the local governments’ behaviors.

## Data Availability

The dataset supporting the conclusions of this article is included within the article.

## Abbreviations

NCMS: The new rural cooperative medical system
RMB: Chinese yuan or Renminbi
GDP: Gross domestic product
GMM: Generalized method of moments
OLS: Ordinary least squares
IV: Instrumental variable

## Footnotes

1 Although the NCMS has been replaced by basic medical insurance system for rural and non-working urban residents, financial subsidies from central government and local governments remain an important part of its funding. Hence, this study based on NCMS data will remain valid.

2 In 2002, the government released the Decision on Further Enhancing Health Services in Rural Areas. Taking into consideration the levels of economic and social development in rural areas, the government decided to drive health services reform to a deeper level, and put in more funding to rural areas, to provide different levels of medical services to rural residents.

3 In fact, by 2016, some provinces had already tried to integrate basic medical insurance for non-working urban residents and the new type of rural cooperative medical care. For example, since the end of 2013, Shandong province has gradually established and implemented a unified basic medical insurance system for non-working urban and rural residents.

4 In 2006, seven ministries, including Health Ministry, jointly issued the Notice on Accelerating the Pilot Programs of the New Type Rural Cooperative Medical Care System, which proposes to expand the pilot areas. In 2006, the number of pilot counties should be reached about 40 percent of the total number of counties; in 2007, it should be expanded to about 60 percent; and NCMS should be covered all rural residents in 2010. To entice farmers to participate in NCMS, the government subsidy standards for NCMS increased from RMB20 at the beginning to RMB200 per person per year in 2011.

5 The first-class regions encompass 12 provinces (autonomous regions and municipalities), including Nei Mongol, Guangxi, Chongqing, Sichuan, Guizhou, Yunnan, Xizang, Shaanxi, Gansu, Qinghai, Ningxia, and Xinjiang; the second-class regions include 10 provinces: Hebei, Shanxi, Jilin, Heilongjiang, Anhui, Jiangxi, Henan, Hubei, Hunan, and Hainan; the third-class regions include 3 provinces: Liaoning, Fujian, and Shandong; and the fourth-class regions are Tianjin, Jiangsu, Zhejiang, and Guangdong.

6 When the central government provides subsidies, the local government shall provide matching subsidies. If the local government fails to provide the matching subsidies timely or the matching funds are insufficient, the central government will reduce the subsidies to the local government correspondingly. The proportion of deduction is usually 10 percent.

7 It’s a simplification of the model, which is assumed that the total central government revenue is used for NCMS subsidies. There are of course many other public services, which we ignore here to keep notation as simple as possible.

8 The detailed calculation procedure is omitted. After adjustment and calculation, it can be obtained that as long as ${\alpha }_{1}\left({m}_{2}{y}_{2}+R+2{\beta }_{1}{m}_{1}{y}_{1}+{m}_{1}{y}_{1}\right)-1<0$, the Eq. (13) is true. As to the validity of ${\alpha }_{1}\left({m}_{2}{y}_{2}+R+2{\beta }_{1}{m}_{1}{y}_{1}+{m}_{1}{y}_{1}\right)-1<0$, when $\left({m}_{2}{y}_{2}+R+2{\beta }_{1}{m}_{1}{y}_{1}+{m}_{1}{y}_{1}\right)<\frac{1}{{\alpha }_{1}}$, it is ture.

9 It is a pity that the “New Rural Cooperative Medical System Statistics” has not been published since 2016. Therefore, data on the number of NCMS participants, central subsidy, local subsidy, and revenue and expenditure are no longer made public.

10 Besides, we have used the tail-shrinking method to eliminate the impact of outliers. After tail-shrinking treatment, the results of the model do not change substantially.

11 The form of the model is based on previous analysis. As mentioned above, the transfer payments expected to receive from central government are related to the numbers of participants, local governments’ matching subsidies, the transfer payments received last year and the development of economic.

12 Hausman test results show that we should choose fixed effect model (*p* = 0.000).
